# The influence of gender norms on post-migration men’s sexual and reproductive health: A scoping review

**DOI:** 10.1371/journal.pone.0322312

**Published:** 2025-08-26

**Authors:** Patience Castleton, Negin Mirzaei Damabi, Mumtaz Begum, Zelalem Mengesha, Zohra S. Lassi

**Affiliations:** 1 Faculty of Health and Medical Sciences, School of Public Health, University of Adelaide, Adelaide, South Australia, Australia; 2 Robinson Research Institute, University of Adelaide, Adelaide, South Australia, Australia; 3 Health Research Institute, University of Canberra, Canberra, Australian Capital Territory, Australia; Caleb University, NIGERIA

## Abstract

Migration exposes people to unfamiliar gender roles and sexual views, influencing the ways they care for their sexual and reproductive health (SRH). Migrant men’s health is overlooked in care and research, prohibiting culturally responsive care that acknowledges these changing beliefs. Thus, we aimed to synthesise the available evidence on the ways pre-established gender norms impact a man’s SRH in the setting of migration to a high-income country. We aimed to investigate how refugee and migrant men viewed gender and gender roles in their countries of resettlement and how they experience their SRH in this country. We systematically searched scientific databases and grey literature (published between 2000–2023), using a search strategy covering four major topics: CALD/migrants, men, gender and SRH. Two reviewers independently screened the titles and abstracts of 7,840 articles and reviewed the full text of 180 articles. Thematic analysis of 36 articles revealed three key themes: (a) depleted masculinity, (ii) sex and sexuality, and (iii) accessing SRH care in a new country, all influencing how migrant men viewed masculinity, engaged in SRH services and approached sexual relationships. Further, navigating new cultural norms and gendered expectations often resulted in feelings of depleted masculinity, impacting men’s wellbeing, intimate relationships and confidence to seek SRH care after migration. We found that migrant men largely carried pre-established beliefs of sex and gender roles from their home country to their host country. This had mainly negative repercussions on their relationships and SRH behaviours post-migration and should be acknowledged in future research and interventions.

## Introduction

Men’s health is a growing public health concern, with men’s life expectancy globally being 5 years lower than women’s [1,2]. Men account for over half of non-communicable disease deaths worldwide [3], and are up to five times more likely to die from cardiovascular disease than women [4]. HIV/AIDS and sexually transmitted infections (STIs) are a leading burden of communicable disease worldwide, with men accounting for over half of sexually transmitted infections globally [5], however they also have poorer access to health care services, particularly sexual and reproductive health (SRH) and mental health [6]. Although data shows that more women are diagnosed with depression, men are significantly less likely to seek professional help for mental health struggles due to masculine ideals and social stigma [7]. Moreover, often due to disruptions in social connections and financial barriers in accessing educational opportunities and healthcare services, poor mental health outcomes are higher amongst migrant populations, including refugees and asylum seekers [8], indicating a higher prevalence amongst refugee and migrant men. As a result, there is a growing global interest in addressing men’s health inequalities through gender-specific health policies. Countries such as Australia, Ireland and Brazil have developed dedicated men’s health policies to tackle these issues [3]. However, due to their health vulnerabilities and low engagement with health services, it is vital that these policies also address the unique needs of men from refugee and migrant backgrounds [9].

As aforementioned, SRH inequalities are more pronounced among refugee and migrant men [4], who experience higher rates of STIs and HIV infections compared to non-migrants and migrant women [10,11]. For instance, nearly half of all male-to-male HIV infections in Australia occur in men from migrant backgrounds [12] a trend also observed in Europe [13,14]. Further, trends in heterosexually acquired HIV infection in high-income countries show a significantly higher number of infections within refugee and migrant men populations compared to their female counterparts [13], and non-migrant men [15]. Despite this, HIV and STI testing has substantially declined in recent years [16,17], with a global study showing over half of the refugees resettled in high-income countries never having been tested [18].

As global migration is increasing, with almost 3.6% of the population classified as migrants, including refugees, skilled migrants and family migrants, health inequalities amongst this population must be a public health priority [19]. By the end of 2023, 117 million people were forcibly displaced, including around 43 million refugees, with men making up roughly half of this vulnerable population [20] and further accounting for almost 60% of all migrants, associated with work, family, and study [21].

Barriers such as financial constraints [18,21], low SRH literacy [18,21] and shame [22] deter men from seeking sexual health testing. Moreover, health inequalities are exacerbated by the often traumatic and unhealthy conditions of the migration journey, exposing men to infection and illness before arrival in their host country [23]. High-income countries host approximately 25% of refugees, and upper-middle countries host 30% [24], with diverse, and often complex, views of health and gender. This presents further challenges to migrant and refugee men accessing quality healthcare in host countries, deterring many from seeking culturally competent services [23].

Current literature on the SRH of refugee and migrant men is limited, but it suggests that these men are less likely to seek medical advice and treatment for sexual health issues compared to both their female counterparts and non-migrant men [25]. This disparity is largely due to traditional gender roles that emphasise strong ideals about masculinity, leading many to view SRH as un-masculine and exclusive for females [26]. Such beliefs hinder their engagement with SRH services and education, both pre-and post-migration, complicating their adaptation to the cultural norms of their host country [27,28]. Additionally, many cultures and religions discourage open communication about sex and sexuality, limiting opportunities to learn from family, community leaders and peers [29]. Men, both migrant and non-migrant, are significantly less likely than women to seek sexual health information from external sources, often due to feelings of shame and embarrassment when discussing sex and sexuality with community members and healthcare providers [12,26]. These feelings are often rooted in the secretive and stigmatised environments they come from, which do not promote open discussions about sex and sexuality [29]. As a result, many men struggle to adapt to the more open communication about sex and sexuality in their host countries, continuing to struggle with discussing their SRH openly.

Current research on refugee and migrant health, particularly SRH, disproportionately focuses on women, leaving significant gaps in our understanding of the health and healthcare needs of men from these backgrounds. It is not well understood how refugee and migrant men navigate their SRH within unfamiliar cultural environments and policies, limiting the cultural sensitivity and responsiveness of services, and thus decreasing their accessibility and use. Additionally, global literature on how these men navigate their gender identity and masculinity in a new country is limited, further reducing the effectiveness of SRH care services and policy. Therefore, we aimed to synthesise the available evidence on the impact that culturally developed views of gender and gender roles have on men’s experience of SRH in the context of migration to a high-income country.

This review was guided by the following research questions:

1. What are refugee and migrant men’s views on gender and gender roles in their countries of resettlement?
2. How do refugee and migrant men experience their SRH in the context of migration to a high-income country?

## Methods

We started by developing a scoping review protocol, following Preferred Reporting Items for Systematic Reviews and Meta-Analysis for Scoping Reviews (PRISMA-ScR) guidelines, which was registered in OSF: https://doi.org/10.17605/OSF.IO/EQ8TG in conducting this scoping review [30] which aimed to answer the research questions detailed above.

### Search strategy

We systematically searched the following eight scientific databases: PubMed, Scopus, Web of Science, Embase, ProQuest (Health and Medical Sciences and Public Health Collections), Emcare, PsycINFO, CINHAL and grey literature (relevant and peer-reviewed reports from organisational websites including the World Health Organisation (WHO), Refugees and Migrants, Amnesty International, The United Nation Refugee Agency, International Organization for Migration, Settlement Services International, Red Cross, Medecins Sans Frontières, Armed Conflict Location and Event Data Project, The Uppsala Conflict Data Program). In addition to these databases, the reference lists of all the articles included in this review were screened to identify further potential articles.

The search strategy (S1 Appendix) was based on four major search topics: Individuals from culturally and linguistically diverse backgrounds (CALD)/migrants (as defined in the Australian context [31]), men, gender and sexual and reproductive health. We included peer-reviewed articles published between 2000–2023, due to the availability of official migration trend data, that adhered to the inclusion criteria detailed in Table 1.

**Table 1:** Inclusion and exclusion criteria of studies included in the scoping review.

	Inclusion	Exclusion when specific
Population	• All individuals who identify as men and from all sexualities (bisexual, heterosexual, gay etc.) who are from migrant, refugee OR asylum seeker backgrounds. • Studies that included men and women but had a subgroup analysis for men.	• Animal studies. • Studies that focus on women only or do not include subset analysis for men.
Exposure	• Studies that examine the experience men from migrant, refugee and asylum seeker backgrounds have in navigating differences in gender stereotypes and cultural beliefs between their home and host country.	• Studies whose primary focus is not on SRH or gender/cultural stereotypes or do not involve men from migrant or refugee backgrounds.
Outcome	• Global studies that examined the SRH needs, care access experiences and outcomes of men from migrant, refugee and asylum seeker backgrounds; including but not limited to abortion, contraception, access to (and experience with) sexual health services, family planning, sexual experience, marriage, fertility concerns, STIs/HIV, sexual/gender-based violence.	
Study designs	• Observational studies (prospective and retrospective cohort studies and cross-sectional studies), experimental (randomised (individually OR cluster) and non-randomized controlled trials including quasi-randomized trials and controlled Before-after (CBA) studies, and original articles and systematic reviews. Qualitative, quantitative and mixed-methods studies will be considered for inclusion. • Studies published in English between 2000–2023. • We will consider studies published from grey literature and academic sources.	• Animal studies, case reports, case series, opinions, editorials, commentaries, PhD/master’s thesis and dissertations, and letters were excluded as well as any article not available in the English language.
Setting	• Studies from any setting (urban and rural areas) where the men have resettled in a high-income country (HIC), as defined by the World Bank [32], from any country of origin. • Studies that include a sub-population of these men will also be included	• Studies that men have resettled in low- and middle-income countries according to the World Bank Classification of countries [32].

### Data extraction and synthesis

All papers identified from the systematic search were downloaded and saved into an Endnote library. They were then transferred to Covidence software for title and abstract screening using the inclusion and exclusion criteria as stated in Table 1.

**Fig 1:**
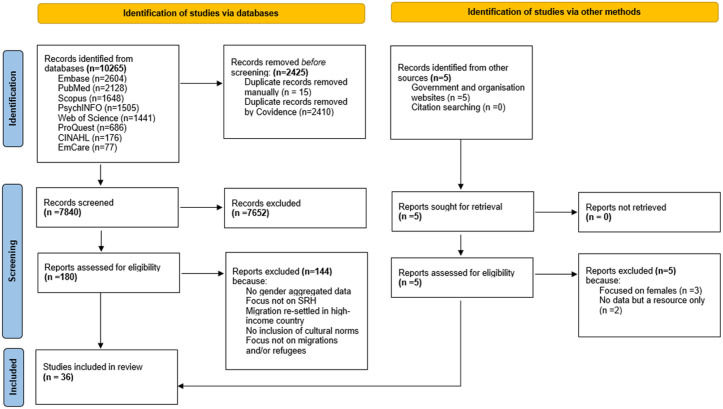
PRISMA flow diagram.

Five authors independently conducted title/abstract screening and full-text review of the articles (on Covidence), and each study was then independently assessed by two authors; results were cross-checked, and differences were discussed and resolved. The PRISMA flowchart (Fig 1) shows the reasons for article exclusion. Basic data was initially extracted from each paper using an Excel spreadsheet developed by the first author, including information on publication year, study design, country of origin and migration and the SRH themes discussed, such as contraception, marriage etc.

**Fig 2:**
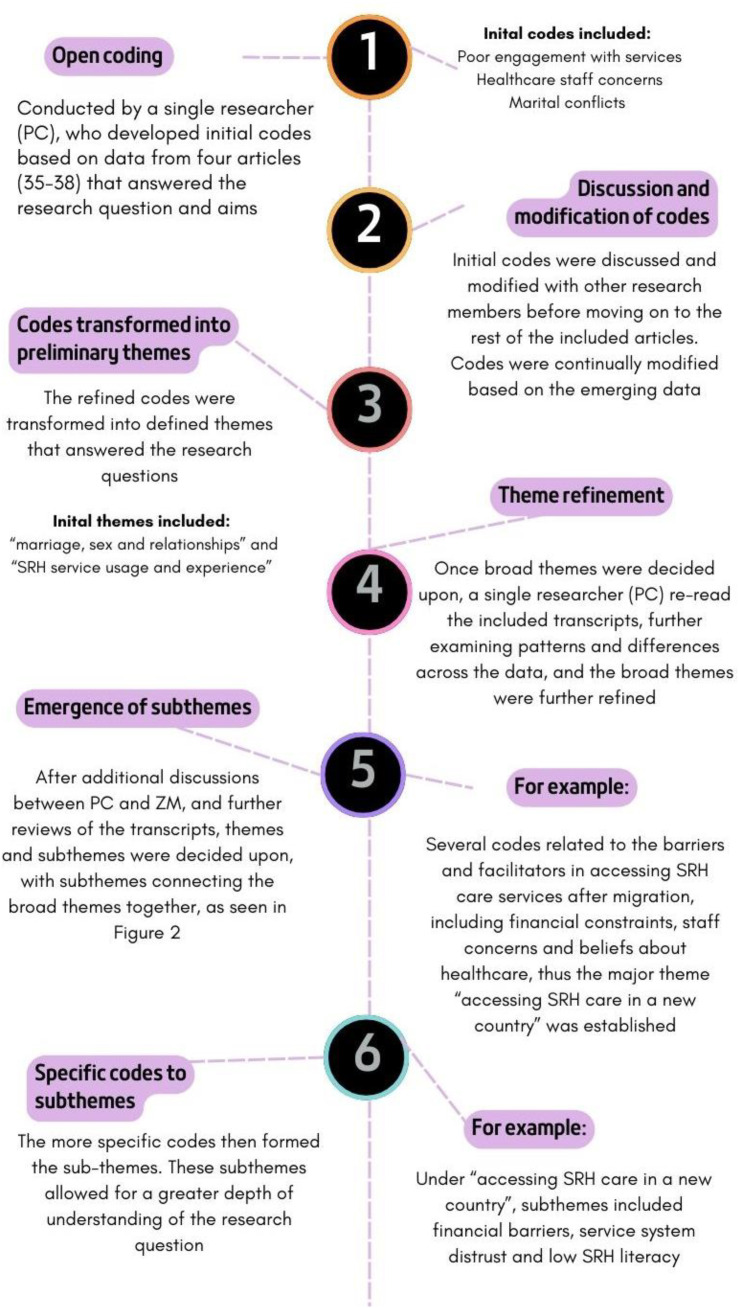
Timeline of thematic coding events.

Due to the high level of qualitative papers included in the review (86.11%), thematic analysis was the most appropriate analysis technique [33]. Frequencies, patterns and relationships found in the quantitative data were transformed into descriptive statistics when thematically analysing the papers. NVIVO software (2023/version 14) was used to analyse and generate initial codes for all included articles, as described in Fig 2. Themes broadly described patterns in the data relevant to the research question.

A theoretical framework developed by Evans et al. in 2011 [34] that explores the influence of masculinity throughout the life course was used to further analyse and interpret the results of this review. The Health, Illness, Men and Masculinities (HIMM) framework was adapted to create a deeper understanding of how masculinity intersects with other social determinants of health, including ethnicity, education and community, to influence health outcomes [34]. The framework acknowledges that social, political and historical contexts, as well as life events, influence a man’s views of masculinity, sexuality and health. The analysis for this review was informed by the HIMM framework of the ways that masculinity intersects with migration and cultural beliefs to impact SRH health-seeking behaviours.

## Results

The systematic search resulted in 10,265 articles, of which 2,425 duplicates were removed. As shown in the PRISMA Flowchart (Fig 1), we screened the titles and abstracts of 7,840 articles, of which 180 articles underwent full-text screening. Our systematic search consisted of grey literature searches as well as scientific databases, however, no eligible studies were found in the grey literature searches. We included 36 articles for final analyses in this review.

The characteristics of the included articles are shown in Table 2. Briefly, we included 32 qualitative studies, 2 quantitative studies and 2 mixed-method studies published between 2002 and 2023. Men included in the studies migrated to North America (n = 12), Australasia (n = 9) and Europe (n = 15), from low/lower-middle, upper-middle income and high-income countries. 8 studies provided data on refugee populations and 24 provided data on migrant populations only, whilst 4 provided separate data off both.

**Table 2:** Characteristics of included studies.

Author, year	Study Type	Data collection type/sample size (n)	Age of participants	Host region/country	Region/country of origin	Gender Included	Migrant Type
Adedimeji,2015 [35]	Qualitative	FGDs (n = 60)	18-64	Europe/ Ireland	Africa	Men and women	Migrants and refugees
Affleck, 2018 [36]	Qualitative	Ethnography & interviews (n = 33)	20-50	North America/ Canada	South Asia/ Sri Lanka	Men only	Migrants
Agu, 2016 [37]	Qualitative	FGDs (n = 45)	18-50	Australasia/ Australia	SE Asia/ Vietnam, Malaysia, Philippines, Indonesia, Brunei and Sub-Sahara Africa/ Burundi, Congo, Ethiopia, Ghana, Kenya, Liberia, Malawi, Namibia, Nigeria, Sudan, The Gambia, Zambia	Men and women (37% male)	Refugees
Ajlan, 2022 [38]	Qualitative	Interviews (n = 14)	32-61	Europe/ Germany	Middle East/ Syria	Men and women (64% male)	Migrants
Baroudi, 2021 [39]	Mixed methods – cross-sectional	Online questionnaire (n = 1773)	16-29	Europe/ Sweden	Middle east, North Africa, South Asia	Men, women and non-binary (59% male)	Migrants
Baroudi, 2023 [40]	Qualitative	Interviews (n = 13)	22-37	Europe/ Sweden	Middle east/ Syria, Yemen, Iran, Palestine, Iraq	Men only	Migrants
Blell, 2018 [41]	Qualitative	Interviews and observations (Phase 1: n = 86 couples, Phase 2: n = 6 couples and n = 6 clinical staff)	NR	Europe/ UK	Middle east/ Pakistan	Men, women and Infertility clinic staff	Migrants
Charsley, 2015 [42]	Qualitative	Interviews (n = 3)	NR	Europe/ UK, Denmark	Middle east/ Pakistan, Turkey	Men only	Migrants
Charsley, 2019 [43]	Mixed-methods– cross sectional	Interviews and FGDs (n = 1, 813 couples with 710 husbands)	20-49	Europe/ UK	Middle east/ Pakistan	Men and women (39% male)	Migrants
Doyal, 2009 [44]	Qualitative	Interviews (n = 46)	18+	Europe/ UK	Africa	Men only	Migrants
Dune, 2017 [45]	Mixed-methods– cross sectional	Online survey (n = 41)	18-39	Australasia/ Australia	Sub-Sahara Africa, SE Asia, East Asia, Eastern Europe, Western Europe, Middle East, The Americas	Men and women (41% male)	Refugees
Fidolini, 2020 [46]	Qualitative	Interviews (n = 35)	25-30	Europe/ Italy, France	North Africa/ Morocco	Men only	Migrants
Fisher, 2013 [47]	Qualitative	FGDs (n = 54) with professionals (n = 24)	18-56	Australasia/ Australia	Africa/ Somalian, Sierra Leonean, Ethiopian, Liberian and Sudanese Communities	Men, women (44% male) and professionals	Refugees
Guo, 2019 [48]	Quantitative	Interviews (n = 506)	18-45	North America/ USA	Asia	Men and women (56% male)	Migrants
Habash, 2023 [49]	Qualitative	Online survey (n = 30 families and n = 20 NGO staff)	NR	Europe/ UK	Middle east/ Syria	Men and women	Migrants and refugees
Hendrick, 2002 [50]	Qualitative	Interviews (n = 55)	15-21	Europe/ Belgium	North Africa	Men and women (49% male)	Migrants
Henrickson, 2015 [51]	Mixed methods– cross sectional	FGDs (n = 131) and survey (n = 703)	16 - > 40	Australasia/ New Zealand	Africa/ Morocco	Men and women (49% male in survey, 41% male in FGDs)	Migrants
Hibbins, 2005 [52]	Qualitative	Interview (n = 40)	19-57	Australasia/ Australia	North Asia/ China	Men only	Refugees
Hirsch, 2009 [53]	Quantitative	Interviews and a follow-up survey (n = 187)	28 years average	North America/ USA	North America/ Mexico	Men only	Refugees, Asylum seekers and Undocumented migrants
Kackowski, 2020 [54]	Qualitative	FGDs (n = 25)	18-24	North America/ USA	Middle east/ Afghanistan, Pakistan Asia/ Burma, Africa/ African Republic, DR Congo, Somalia South America/ Colombia	Men and women (52% male)	Migrants and refugees
Keygnaert, 2014 [55]	Qualitative	Interview (n = 223)		Europe/ Belgium, Netherlands	Middle east/ Iran, Iraq, Somali, Afghanistan	Men and women (39% male)	Migrants
Kingori, 2018 [56]	Qualitative	Interviews (n = 27)	18-25	North America/ USA	East Africa/ Somali	Men and women (48% male)	Migrants
Maternowska, 2010 [57]	Qualitative	Interviews (n = 44)	17-25	North America/ USA	North America/ Mexico	Men and women (40% male)	Migrants
Maternowska, 2014 [58]	Qualitative	Interviews (n = 23)	19-37	North America/ USA	North America/ Mexico	Men only	Migrants
McKeown, 2010 [59]	Qualitative	Email survey (n = 112)	18-52	Europe/ UK	The Americas/ Caribbean, Africa, Middle east/ Pakistan, South Asia/ India	Men only	Refugees
Mole, 2014 [60]	Qualitative	Interviews (n = 17)	25-40	Europe/ UK	Central and Eastern Europe/ Lithuania, Poland	Men only	Refugees
Muchoki, 2015 [61]	Qualitative	Interviews and FDGs (n = 18 men and n = 7 informants)	24-75	Australasia/ Australia	Africa/ Sudan, Eritrea, Ethiopia, Somalia	Men only	Migrants
Nordström, 2021 [62]	Qualitative	Interviews (n = 8)	18-22	Europe/ Sweden	Middle East/ Afghanistan, East Africa/ Somalia, Yemen	Men only	Migrants
Oliffe, 2007 [63]	Qualitative	Observations (n = 14)	63-88	North America/ Canada	South Asia	Men only	Migrants
Phillpot, 2023 [64]	Qualitative	Interviews (n = 24)	19-64	Australasia/ Australia	South and SE Asia/ Philippines, Thailand, Vietnam, Malaysia, Cambodia, India, South America/ Colombia, Ecuador, Peru, Brazil North America/ Mexico, East Asia/ China, Taiwan, South Kora, Europe/ Russia	Men only	Refugees
Rhodes, 2009 [65]	Qualitative	Photovoice (n = 9)	18–29	North America/ USA	North and Central America/ Mexico, El Salvador, Guatemala	Men only	Migrants
Russo, 2023 [66]	Qualitative	Interviews and FGDs (n = 57)	22-49	Australasia/ Australia	Middle East/ Afghanistan	Men and women (50% male)	Migrants
Satyen, 2020 [67]	Qualitative	FGDs (n = 37)	41-80	Australasia/ Australia	East Asia/ China	Men and women (54% male)	Migrants
Shedlin, 2006 [68]	Qualitative	Interviews, FGDs and ethnographic observations (n = NR)	NR	North America/ USA	South Asia/ India, West India/ Caribbean North and Central America/ Mexico	Men and health providers	Migrants
Stephens, 2017 [69]	Qualitative	Interviews (n = 45)	18-25	North America/ USA	Central and South America/ Mexico, Nicaragua, American Argentina, Venezuela, Colombia, Other Hispanic	Men only	Migrants and refugees
Winett, 2011 [70]	Qualitative	Interviews (n = 49)	18-30	North America/ USA	North America/ Mexico	men only	Migrants

SE Aisa = Southeast Asia, USA = United States of America, UK = United Kingdom, FGDs = Focus Group Discussions, NR = not reported

**Fig 3:**
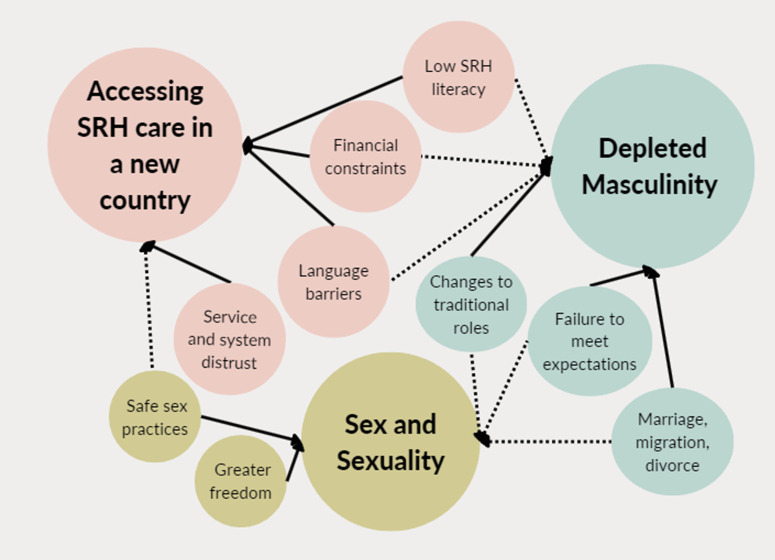
Themes emerged through thematic analysis of included studies.

Through thematic analysis, three major themes emerged based on our research question, (i) depleted masculinity, (ii) sex and sexuality and (iii) accessing SRH care in a new country. These themes set a framework behind how and why migrant men interacted with SRH care services after migration and the impact that migration had on their views of themselves as men. Information from these themes was further thematically analysed to defined subthemes, further explaining the connections between migration, masculinity and SRH, as detailed below and in Fig 3.

### I. Depleted masculinity

This theme discusses the ways in which the differences in cultural beliefs and practices impact refugee and migrant men’s views of gender and how these changes result in feelings of depleted masculinity.

(i) Changes to traditional roles: “lack of domestic authority”

Many studies in this review found that refugee and migrant men struggled to understand and accept the “threatening” [58] change in “gender and power dynamics” [58] after migration [38,42,43,47,49,52,58,61,65,67], with many Asian and black men living in families where the household is the mothers domain and everything outside of the household is the fathers [58]. However, as many host countries do not follow these traditional gender roles, some men, from a wide range of cultural backgrounds, perceived changes in their gendered responsibilities as “going against religion and tradition” [38] and believed their wives were “too confrontational” [49] and “outspoken” [68] in their new country. However, some men from Asian, black and Hispanic backgrounds, believed “there’s no specific difference in what a man or a woman can do” [67] and felt the change to be “empowering” [58]. This sense of depleted masculinity mainly stemmed from men from global backgrounds no longer being the “head of the house” [47] and losing their “breadwinner status domestically” [43]. This forced men to learn new domestic skills, previously attributed to being “woman’s duties” [57,58,65,68], which caused many to feel “powerless and frustrated” [65]. Two studies involving men from Hispanic and Mexican backgrounds in this review similarly reported that men participated in these domestic duties in their new country but stopped in front of their community, feeling shame with their perceived decreased domestic status [57,68]. In contrast, others from Pakistan found “alternative but valued forms of masculine identity… far removed from the oppressive patriarchal figures”, mainly in “being responsible and caring husbands” [42]. One study involving Mexican men in the United States of America reported that, whilst the “conservative nature of their expectations around gender roles”, including helping with the house work, was maintained after migration, many men did begin to participate in duties they deemed to be “the work of women” [57]. Thus, many found these migratory changes in cultural norms to be a protective factor, with men developing “increased respect of their wives as equal partners” [58].

Moreover, refugee and migrant men across studies strongly believed that women were “more suited for taking care of children” [49,52,67], a belief that was not influenced by the duration of settlement in one study [52] but was deeply ingrained in their cultural beliefs and norms. This “lack of domestic authority” [42], often combined with unemployment and loneliness, resulted in poor mental health among many husbands from diverse cultural backgrounds, contributing to negative experiences in their new country [42,43,47].

(ii) Failure to meet expectations: “inadequacy, helplessness and failure”

Eight studies in this review found that feelings of cultural “inadequacy, helplessness and failure” [36] was a source of depleted masculinity for refugee and migrant men [36,40,41,46,52,58,65,69]. Many cultural beliefs showed men, particularly those from Mexican and African backgrounds, as the “provider, protector and guardian” of the family, which “implied the powerful status of men” [52] and displayed their “predatory sexuality” [46]. Thus, refugee and migrant men who failed to build this traditional family (a wife and multiple children), were unable to prove their “legitimate heteronormative sexuality” [46] for both their families in their home country and others in their new country [40,41,46,58,61].

In contrast, some studies involving men from Mexican and Afghanistan, found that “sexual banter and conversations” [52] were a greater source of masculine validity within their new culture [52,65]. Thus, some were able to maintain their masculine status through engagement in these conversations, regardless of relationship status or sexual activity. Further, many men from diverse cultural backgrounds felt increased pressures to build a family after migration, some reporting comments of “you’re worthless, you’re useless” from family members when struggling to meet family and cultural expectations of a “big family” [44,57]. Studies further reported that men from Hispanic and Mexican origins maintained ideas of “machoism”, “infidelity” [58,69], non-use of contraception [58], and “always want[ing] to initiative sex” after migration as ways to “prove their masculinity” through sexuality [69].

Additionally, the unemployment that many refugee and migrant men faced often resulted in them feeling they had lost their “credibility” as a man and a provider [36,43,44,46,47,63,65,67,70]. This resulted in some men, particularly from Afghanistan and Hispanic backgrounds, seeking “unstable sexual partnerships” to gain “housing and support” and a way to compensate for the masculinity they felt they had lost [65,68]. However, unemployment also led to feelings of shame and emasculation for many men due to their inability to support themselves and their families. This depleted some Hispanic men’s “sex drives, motivation, activeness and involvement with their duties” [68], common effects of depression.

The analysis also identified that some men’s feelings of cultural and masculine “failure and inadequacy” were exacerbated by “negative comments from their wives”, “shaming” and criticizing their “lack of economic opportunity” [36]. Some men, from a range of cultural backgrounds including Moroccan and Asian, reported “devising strategies” to reclaim “their power” [61] and “leadership” [49] over their household, turning to anger and violence towards their wives. In contrast, a mixed-methods study found that men who migrated from Africa after their wives struggled with their in-laws’ ability to “exploit their position of power” and force them into domestic chores as ways to undermine them [43]. This resulted in “tension with their own gendered aspirations for life post-migration”, exacerbating feelings of depleted masculinity [43]. Further, some men reported that their wives contributed to these feelings of “emasculation” by supervising their domestic duties, an already threatening chore for many of these men, further resulting in resentment toward their wives and their life in their new country [43].

(iii) Marriage, migration and divorce: “against our traditions and customs”

Six studies included in this review involved married and/or divorced migrant men with conflicting views on marriage and divorce [36,38,42,47,61,67]. For some migrant men from a number of cultural backgrounds, including Asian and Middle-Eastern, divorce was seen as going “against our traditions and customs” [36,38], many blaming migration and the changes in gender norms for the deterioration of their marriage. Further, some men claimed divorce after migration was an “exploitation of the situation” by their wives, whose migration was funded by their husbands and who could file for a divorce immediately after migrating [38]. Studies showed that many refugee and migrant men from Middle-Eastern, Hispanic and Mexican backgrounds believed the legal and social systems in their new country were “inclined towards women” [67], believing that negative representations of themselves by their ex-wives would be “easily accepted by institutions of state” [42]. Thus, many questioned if laws around divorce and conflict were “rational” [38,47,61,67], causing feelings of inferiority and depleted masculinity. One study reported migrant men from Pakistan being treated as if they “are zero” and “an America’s movie villain” [42]. Moreover, after migration, many Asian men felt that the unfamiliar “formal systemic response” of officials was “affecting our relationship and our family”, by always looking “at the side of the woman” in situations of marital conflict [47]. As most men were used to conflict being resolved in private settings where they had superiority, this was seen as “symbolising men’s disadvantaged position”, which contributed to feelings of depleted masculinity and anger of migrating [47].

### II. Sex and sexuality

This theme explores the ways in which views and understanding of sex and sexuality change after migration, the experiences refugee and migrant men have and how they navigate these changes.

(i) Greater freedom: “experience their masculinity through an active sexuality”

Greater sexual freedom often came with migration, with men’s new countries seen as more “permissive” societies [61], having “more liberal attitudes” [48] towards SRH [45,48,50,52,55,59–62,64,66,68,70]. Many appreciated the country’s sexual freedom, wanting to “move away from the cultural silencing of sexuality” [45,66], enabling them to explore greater sexual opportunities and learn more about SRH. However, some Middle-Eastern men also felt that Western countries “view migrant ideas of SRH as out-dated”, arguing that their views are not as “traditional” and “taboo” as many believe [45]. An included quantitative study found that both American’s and Asian migrants held similar views regarding sexual initiative, wife choosing and sexual freedom, even reporting less conservative judgements of female sexual behaviours than white Americans, thus supporting the idea of “out-dated” migrant ideas [48]. The stigma and racial discrimination perpetuated towards them within these beliefs decreased their confidence and comfort in their new home [45].

Many included studies commented on migrant men’s post-migration opportunities to have “casual or compensated sexual relationships” [61], including sex with multiple sexual partners and sex workers. Most men saw this as a “positive or neutral” change, enjoying their new sexual freedom [48,50,53,58,60,61,66,68,70]. Thirteen studies correlated this freedom to men living away from their families as this “reduced the likelihood of sexual activities being discovered” [61] by disapproving family members and cultures [45,48,50,52,55,59,60,62,64,66,68,70]. However, this was controversial for some men in seven studies who mentioned that they felt “morally bound” [45] and “limited” [39] by their cultural beliefs and family. Thus, many sought to avoid community “disapproval” by limiting their exploration of sex and sexuality [39,45,46,62,66,70]. Further, promiscuous sexual experiences came with religious guilt for some migrant men, from South Asian backgrounds, who lost connection to their religion after new sexual experiences; “I don’t feel like a Muslim now” [62]. Some men, from multiple cultural backgrounds including Asia and the Middle-East, also viewed this greater freedom as a new societal pressure, causing them to “doubt one’s own masculinity and heterosexuality” [46] if they “reject the advances of young women” [46,50,52,54,66]. Thus, many conformed to these norms even if they did not want to for fear of losing their masculinity and wishing to fit in with their new country. This finding was aligned with data from a mixed-method study that showed almost 15% of Arabic-speaking migrant men feeling dissatisfaction with their sexual life [39]. Further, one study found that migrant men from African backgrounds wanted to “experience their masculinity through an active sexuality that does not wait for marriage”, seeing migration as an opportunity to defy cultural obligations of abstinence [46]. Seven studies further commented on the sexual activities of men separated from their parents after migration, claiming it to be “a necessity” [70] and something they would happily do, and would need to do, after migration, even if infidelity was against their cultural beliefs [40,48,53,60,66,70]. Some refugee and migrant men from diverse cultural backgrounds “simultaneously saw inﬁdelity as both wrong and normal” [70], alluding that, whilst it goes against their culture and beliefs, it is something they engaged with after migration. This was supported by one of the included quantitative studies, involving men from a range of cultural backgrounds including South America and Africa, that saw positive relationships between migration induced family separation and increased extramarital sexual partners [53].

Holding a woman’s virginity in high regard was still common amongst many migrant men, with some wanting to marry a woman with no previous sexual partners [48,61]. This caused many, particularly those from the Middle-East and Africa, to seek “a marriage partner overseas” (commonly in their home country) as they are “less promiscuous” than the women in their new country [61]. Some men from East Asian backgrounds also negatively commented on migrant women’s abilities to have multiple sexual partners, claiming the “culture is very bad for men, but it is good for women because here, women are gaining control” [66]. Many men from a range of Middle-Eastern and African backgrounds saw woman’s sexual freedom as a threat to their masculine dominance, whilst simultaneously enjoying the freedom for themselves [61]. Conversely, some men’s acculturation experience resulted in an increased “recognition of women as equal and valued partners within sexual encounters” [66] and did not feel their masculinity was jeopardised by women’s abilities to have liberal sexual experiences [48,66].

Ten studies included in this review found that migrant men were able to “talk about sex freely” [40] in their host country [37,40,45,50,54–56,60,62,66]. Many men from Middle-Eastern backgrounds found that “discussion of sexual health issues was “more acceptable” than in their home countries and were “appreciative of the exceptional knowledge levels” that doctors and educators had of SRH issues [37]. Many migrant men were raised in Middle-Eastern and Mexican communities where sexual health problems were viewed as “a threat to masculinity”, thus conversations of SRH were “accompanied with shyness, shame, and taboo” [40], with some fearing “being ostracized” if they mentioned sex in their home country [56]. Thus, the “lingering effects of the traditional system” continued to impact some men’s abilities to speak “openly about sexuality” [60] even after migration [40,50,54,56,62].

(ii) Safe sex practices: “struggled to adapt [to] unfamiliar sexual norms”

Many refugee and migrant men “struggled to adapt [to] unfamiliar sexual norms” [64], including the use of contraception and initiation of intercourse [39,51,54,57,58,68]. Men commonly held negative associations with condoms; largely attributed to traditional and cultural beliefs and low SRH literacy. Some South Asian men’s religious beliefs “oppose birth control” and were thus never educated on its use and importance and were thus never educated on its use and importance [51]. Others from Middle-Eastern and Latin American backgrounds believed condoms “interfere with men’s virility and sexual performance” [57,58], and are “not associated with pure love” [68]. Further, some mentioned that “condom-less sex” was “far more normative” in their host country as compared to their home, leading them “to practice the same as others do” [64]. Many from African backgrounds understood that “using contraception in general” ensured “good sexual health” [50,55] and had practiced safe sex in both their home and host countries. Additionally, one study involving migrant men from Mexico mentioned that after migration, men felt they were “more responsible” to use contraception, thus increasing their use of condoms [57].

### III. Accessing SRH care in a new country

This theme presents a summary of the barriers and facilitators refugee and migrant men experience in accessing SRH countries in their new countries of resettlement.

(i) Financial constraints: “more willing to use such facilities if such services were reasonably priced”

Studies included in this review identified that worldwide, refugee and migrant men faced “financial challenges” [35] when seeking support from SRH care services [35,39,43,44,54,63,64,67], prohibiting them from accessing SRH care, and negatively impacting their health outcomes after migration. Although some studies including men from a range of cultural backgrounds, including Afghanistan and Latin America, reported that refugee and migrant men were satisfied with their new employment opportunities and financial status [58,65], others identified men as having “low-paying jobs” [35], often “below their original qualifications’‘ [44], and were thus “unwilling” to pay for health services “because of other commitments” [35,43,67]. Men agreed that they would be “more willing to use such facilities if they were reasonably priced” [35,54].

Further, many studies identified financial barriers to gaining medical insurance cover [35,37,68], accessing transportation to attend appointments and continuing with care advice, including paying for medications, contraception [54,63] and medical tests [54,63]. The low financial security of many refugee and migrant men from Middle Eastern backgrounds had further implications on their ability to “do anything else”, including attending English language lessons, and learning new skills for employment opportunities [41], negatively influencing their abilities to receive and understand health care advice given.

(ii) Language barriers: “trapped by their poor English proficiency”

Low English language proficiency was a barrier to accessing SRH care and education for many refugee and migrant men [35,37,39–41,43,54,63,65,68], some reporting feeling “trapped by their poor English proficiency” [41]. However, studies involving men from multiple backgrounds, including Asia, the Middle-East and South America, further commented on the use of translators during SRH consultations, to which more men agreed did not “guarantee effective communication” [63]. Some men were “uncertain about the reliability of interpreting” [40] and had concerns about the confidentiality of their information with a translator [40,63]. Thus, translators did not always serve as an effective incentive for refugee and migrant men to seek SRH care.

Whilst some studies reported men were able to speak with new health providers without feeling “shy” [40], many men lacked the ability to understand the information presented to them in consults and/or promotional material [37,39,40,50,51,54,56,60,63–65,68]. Their “limited confidence to engage in conversation” [43] with English speakers limited them from attending appointments with English-speaking staff. This struggle was further exacerbated by men’s inability to “decode public transport information” to attend appointments and navigate English phone/online booking systems [43], therefore, were unable to make and attend an appointment regardless of their health symptoms.

(iii) Low SRH literacy: “no words in their mother tongue for SRH”

Low SRH literacy and understanding of available services emerged as an important barriers in men from refugee and migrant backgrounds’ confidence to seek SRH care [37,40,50,51,54,56,60,63,64,68]. The cultural “negative connotations” [37] surrounding sexual health [37,56,64] and the limited SRH education received in their home countries, which included countries in the Middle East and Latin America, by both institutions and families [40,56], resulted in many refugee and migrant men having their first “exposure to these topics” [37] after migration.

Men’s knowledge of key SRH topics, including contraception, STIs and safe sex, differed greatly depending on their country of origin, education levels and community, as explained by the HIMM framework [34]. Some studies found sound knowledge of AIDS and HIV among migrant men [50,65], whilst others found men to have limited or incorrect beliefs about their SRH illnesses [60,63,68]. Knowledge of contraception [50,54,56] and safe sex [60] was also reportedly low, with religious beliefs and cultural laws playing a large role in their prior education of sexual practices. One study, focusing on men from the Middle East and Pakistan, also reported that, as the men had not received SRH education in their home countries, but migrated at an older age, they were responsible for “studying these things alone” [40], which was done by many using the Internet as an information source [40,54,56]. Whilst this would have been acceptable for some, others may not have internet access and may not have the language and/or computer literacy skills to retrieve and understand information. For example, one study showed that migrant men from Middle Eastern countries considered sexual health to relate only to infertility and reflected on their home countries belief that “sexual satisfaction and pleasure were not considered as important as building a family” [40]. These men would, thus not know what further SRH information is needed to expand their SRH vocabulary and may have difficulties understanding and relating to SRH topics. Furthermore, some migrant men, mainly from North Africa and the Middle East, found they had “no words in their mother tongue for SRH” [45] terms, making it difficult to interpret their meaning even with a basic understanding of the English language, thus disincentivising them from educating themselves and seeking SRH care [37,45,54].

Many studies reported men had a “lack of awareness” of service centres [37,39,57,65], including testing for HIV and STIs [35,64,65] and family planning [57]. Interestingly, a mixed-methods study found that almost 40% of studied men from a range of Middle-Eastern backgrounds, including Syria and Iran, didn’t know where to gain SRH knowledge after migration, whilst only 30% of females didn’t know where to get SRH information [39]. Some Middle Eastern men attributed this lack of knowledge to the “low frequency of messaging” [37] in their host country regarding available services. Many finding it “harder” to receive information on SRH, including “information about AIDS and condoms”, and care services after migration compared to in their home country [65]. This resulted in both low knowledge of where to seek help and insufficient reinforcement that these care services are necessary and available. This could be further exacerbated by the low SRH literacy and English language proficiency of many men from refugee and migrant backgrounds. Included studies further found that migrant men from North and Central American backgrounds considered SRH services to be “women’s services” [57] as they were “not very seen” in their home country, leaving them unaware of their availability and importance [64]. Most of these studies reported that migrant men highly appreciated the “availability of sex education in [migrant countries] schools” and that the open communication of their host country “increased their awareness of the treatments that are available to achieve this [optimal sexual health and performance]” [37,40,62,66].

Knowledge of contraception [54] and safe sex [60] was also reportedly low, with religious beliefs and cultural laws playing a large role in their prior education of sexual practices. Many men, mainly from the Middle East and Latin America, associated condoms with pregnancy only, disregarding infection, or not perceiving “risk [of STI] from girlfriends, with no acknowledgement that they could be infecting their female partners” [39,68]. Further, others from South Asian backgrounds held the stereotypical belief that they were “out of the environment” of HIV [51] after migration, thus feeling they did not need to protect themselves from it as it was not apparent in their new country. However, some refugee and migrant men believed they were at higher risk of HIV in their new country due to “greater ethnic diversity and increased sexual activity” [54,60,68]. One study focusing on African men mentioned that refugee and migrant men used condoms when engaging in “casual relationships and in contact with a prostitute” seeing these activities as a higher risk of infection [50].

(iv) Service and system distrust: “You are better off doing something on your own”

Many included studies indicated that distrust in the host country’s health services and health system were barriers to accessing SRH care [35,37,39,40,54,63,64,68]. Men from refugee and migrant backgrounds commonly found that SRH care in their new country was “different” to that of their origin country [45,54], with contrasting critiques of the systems. A major concern was the “tedious” [37] long wait times to see a doctor [35,39,40,63]. As a result, many did not have the time or patience to visit a health professional for SRH issues, particularly those who viewed SRH as a low priority compared to other challenges, including employment. Some men believed they were “better off doing something on your own” as they didn’t have time to wait for a “consultant or specialist” [35]. Further, some men, from a range of backgrounds including South Asia, East Asi and South America, did not understand the concept of patient “waiting lists” for procedures such as elective surgery and often interpreted this as “a doctor’s incompetence”, as this is not something they would experience in their home country [63]. However, others reported positive service experiences, trusting the care they were receiving was satisfactory and confidential and feeling “respected, listened to and cared for in SRH services” [40,45]. Greater “openness” and “trust” in the services came with longer settlement times in one study that focused on men from the Middle-East in Sweden [40].

A major concern for others was in the confidentiality of their information with doctors [35,37,39,54]. Young men from Middle-Eastern backgrounds, in particular, stated they were “not allowed to talk about that [sex]”, and feared their community would find out about their sexual activity if they consulted a doctor [54]. Further, some North African men felt SRH care workers were “minimally equipped to deal” with their unique needs [45] and were thus sceptical to seek consults and trust the advice given. In contrast, others acknowledged that it would be “hard” for healthcare workers “to keep up” [45] with the varying SRH messages and needs of migrant populations. Many of these navigation difficulties seemed to stem from Middle-Eastern men’s visa and residency status [37,42], some fearing “visa-refusal” if they were sexually ill [37]. Many did not trust the health care services and the confidentiality of their consults [35,37,39,54], these visa fears further prohibited them from discussing their SRH with others and seeking any professional care.

Studies in this review also indicated that the “discrimination” [37] and “underestimation” [40] perpetrated toward migrant men by health care services stopped them from trusting in medical advice, deterring them from attending consultations [35,37,39–41,45,54]. This enhanced many men’s desires to seek SRH care from a provider from the same race/religion as themselves, believing their health advice is “less likely to be biased since we are from the same continent” [41,63,64]. Alongside the ability to converse in their language, men from a range of backgrounds including South and East Asia, South America and Russia believed health professionals from similar backgrounds were able to “understand [them] as a person as well as a patient” [63] providing no racial bias. However, many found it difficult to identify these professionals in their local area, deterring them from accessing any SRH services [63].

## Discussion

The purpose of this scoping review was to consolidate the available global literature that details the impact that culturally developed views of gender and gender roles have on men from migrant and refugee background’s experience of SRH. Thematic analysis of 36 articles revealed three major themes that connected migration, masculinity and SRH: (i) depleted masculinity, (ii) sex and intimacy and (iii) accessing SRH care in a new country. The HIMM framework [34] helped to demonstrate the connections between these themes and enhance our understanding of masculinities as social determinants of health.

Our review found that migration often resulted in feelings of depleted masculinity due largely to changes in traditional roles, employment status and feelings of cultural failure. This finding aligns with previous research that similarly identified men from refugee backgrounds feeling their masculine power is lost after migration [71]. These feelings were often exacerbated by precarious employment and financial struggles, causing men to place a large pressure on emphasising their masculine roles in their new county [71]. Additionally, we found that navigating new cultures of sexuality, and sexual health, was a significant challenge for many refugee and migrant men. Previous literature has similarly shown that refugee and migrant men had to adjust to different contraceptive preferences and practices, family planning methods and ways of pursuing intimate relationships after migration [72,73].The current review, and previous literature, similarly showed that this caused significant adjustments to many men as they struggled with new SRH concepts and practices, especially for those with low SRH literacy. Finally, our review identified low SRH literacy, language barriers and financial constraints to be significant barriers in men’s abilities to access SRH care services after migration. These barriers are evidenced in previous literature that shows low uptake of SRH care services, including accessing HIV testing amongst refugee and migrant men [74,75].

Based on the findings from this review, and those shown in previous literature, comprehensive, culturally appropriate SRH education must be provided to all men immediately after migration [15,76,77]. As shown in the current review and in previous literature, no one cultures sexual practices are necessarily negative or positive, it is however, important for refugee and migrant men to be comfortable and able to explore their SRH and sexuality after migration. Therefore, this SRH education must be presented in a respectful manner that does not diminish any one person’s beliefs or practices but instead empowers them to explore their new countries standard SRH practices and services.

The high prevalence of low SRH literacy amongst these vulnerable men must be foremost addressed during initial health examination and/or civic orientations when first entering their new country. This immediate and official presentation of vital SRH information will assist in creating a safe and comfortable environment in which men can gain information on their SRH, thus empowering their use of SRH services [77]. Due to the commonly experienced concerns surrounding confidentiality with general practitioners, studies recommend that education is provided by male professionals who are well-trusted by the community [15]. Research shows that integrating literacy, culture and language with education provides a comfortable and trusted environment to enhance engagement and learning outcomes for refugee and migrant men [77]. Thus, it is important that professionals assess the literacy capabilities of men before providing SRH information. Further, migrant health programmes in Sweden have shown that both gender-separate and mixed education groups are important for comfortable information sharing and discussions of sexual health norms and gender [78]. As identified in this review, knowledge of contraception, healthy relationships and sexual health services and systems is low amongst many migrant men [78].Thus, it is important to integrate these topics in SRH education programs to provide basic, essential knowledge and empower confidence in their sexual health and help-seeking behaviours. Moreover, it is evident that education around gender norms and roles is essential in empowering men to challenge norms of masculinity and domestic power [76].

As aforementioned, rates of refugee and migrant employment are low globally, despite their skills and prior experience, presenting a significant financial barrier in refugee and migrant men’s engagement with SRH services and contributing to feelings of depleted masculinity. As shown in our review, and supported by current literature [79], migration can cause men to find employment in sectors outside of their experience and knowledge, including in roles with unstable working hours and low income. Literature has shown that these precarious working conditions have a negative impact on health and wellbeing outcomes [80,81]. These unfavourable working hours and conditions also impose limitations on refugee and migrant men to carry out domestic care work or seek preventative health care. This implies that promoting diversity and awareness within both business and policy is essential to improve rates of refugee and migrant employment in sectors with greater stability [82,83]. Previous research, aligning with the findings of this review, shows that low proficiency in the language of the host country hinders refugee and migrant men’s abilities to secure stable employment [84,85]. Empowering industry to incorporate pre-employment programs with refugee and migrant men could help to improve English proficiency and understanding of workplace expectations, especially in the context of English speaking settings [82,83]. Thus, providing employers with comfort and confidence in their workers. Further, promoting success stories and creating a positive narrative of refugee and migrant employment within the industry and in media would assist in encouraging employers to seek these men for future opportunities [83]. Newcomer support organisations, such as those that exist in North America, have proven helpful in assisting migrants with work opportunities, and career expectations and fostering hope for the future [82]. However, barriers to these organisations’ abilities to provide support exist largely due to a lack of funding and awareness [82]. Thus, legislation should prioritise financial assistance to these organisations and information on their services should be provided to migrants immediately upon entrance to their new country.

Finally, our review showed the cultural responsiveness of host country health systems needs improvement to better encourage and welcome refugee and migrant men. Enhancing cultural competence amongst healthcare services through practitioner student education programs, position statements and advocacy programs has previously proven successful in providing and promoting culturally congruent care [86]. Thus, policy should invest in improving cultural competency within SRH care systems, ensuring all practitioners are equipped with skills and knowledge to discuss sensitive topics with men from refugee and migrant backgrounds. Further, it is imperative that SRH information is presented in translated materials, enabling all men to access and understand the information [87]. In agreement with previous literature, our review found that many refugee and migrant men were more engaged with SRH services when provided by a male professional, from a similar cultural background [11,63]. Thus, it is important that cultural diversity is evidenced and promoted in SRH care systems. Specialised services for refugee and migrant populations have previously shown success in empowering health service use by providing in-depth information on health systems, assisting in the navigation of the unfamiliar system [88]. Expanding these services and connecting them with key stakeholders and other health services could enhance the cultural competence of mainstream health services, building trust between the system and refugee and migrant men [88].

The findings of this review also carry implications for future research on refugee and migrant men’s SRH. Most of the studies included in this review focused on three key topics of the WHO SRH framework: prevention and control of HIV and other STIs and contraception counselling and provision [89]. Other key topics, including sexual function and gender-based violence prevention, were not addressed and should be the focus of future research in this area. Further, discussing SRH service use and delivery with healthcare providers and men from refugee and migrant backgrounds will assist in gaining a greater understanding of how to enhance culturally competent services. Thus, future research should incorporate first-hand insights from consumers and providers, through participatory and longitudinal research studies, to understand the barriers and facilitators in seeking and providing SRH information and care after migration. Understanding how, and why, some men have positive migration experiences and outlooks on SRH and gender, could help to guide, and improve, the experiences of other men in a new country. This insight would further guide policy change to better respond to the unique, and changing, SRH care needs of men from refugee and migrant backgrounds.

The HIMM framework [34] showed how masculinity intersects with health to influence SRH help-seeking behaviours post-migration. The framework strongly aligned with the themes revealed in our review, providing further depth to the connections between masculine views and SRH outcomes. However, the framework focuses on how masculinity and health change over the life course and does not acknowledge the changing migration status of some men and how this may impact their social determinants of health. Thus, whilst the HIMM framework is helpful in connecting masculinity and health, future research analysing the health and wellbeing of men from migrant and refugee backgrounds should incorporate additional considerations and frameworks specific to migration patterns.

### Strengths and limitation

This review has some strengths and limitations that should be acknowledged. To the best of our understanding, this is the first global scoping review to synthesise the available evidence on the impact that culturally developed views of gender and gender roles have on men’s experience of SRH in the context of migration to a high-income country. Further, our global review included refugee and migrant men from over 25 countries, providing an excellent understanding of the diverse beliefs and SRH practices of men from a wide range of backgrounds. Similarly, as discussed above with the HIMM framework, it is important to note that ‘migrant’ and ‘refugee’ men are not necessarily similar in their migration journeys and background, thus resulting in different views of masculinity and SRH. Despite this, studies included in this review did not disaggregate results by refugee status, thus making it difficult for our review to aggregate based on different migrant groups. Furthermore, the vast majority of included studies were qualitative in nature, thus urging for future research to include more quantitative and mixed-methods approaches. Whilst this study focused only on those migrating to high-income countries, limiting the reach of more global studies, only three geographic regions were recognised in the included studies (North America, Europe and Australasia). Future research should include a broader range of migration context to deepen our understanding of how diverse cultural backgrounds shape migration experiences and sexual health across different host countries.

## Conclusions

Through thematic analysis of 36 qualitative, quantitative and mixed-methods articles, we found that connections between depleted masculinity, SRH care access after migration, and sex and intimacy are heavily influenced by the cultural norms of gender and sex in refugee and migrant men’s origin countries. The connection of these three key ideas shows that policy and SRH care must encompass greater culturally relevant and competent care and education for migrant men. SRH information, encompassing SRH services and systems, contraception and gender and sex norms, should be incorporated into initial information packages and health testing post-migration. Providing this information in a culturally appropriate manner upon arrival will empower men to seek further care and information and promote open communication around SRH.

## Supporting information

S1 Appendix Search strategy: (population) (men) (gender Norms/stereotypes) (sexual/reproductive health). (DOCX)

S1 File (DOCX)
