# Supplementary material for: The influence of gender norms on post-migration men’s sexual and reproductive health: A scoping review
Source: PLoS One. 2025 Aug 26;20(8):e0322312. doi: 10.1371/journal.pone.0322312 (PMC12380284; doi:10.1371/journal.pone.0322312)
Supplement: S1 Appendix — (DOCX) [file pone.0322312.s001.docx]

**SEARCH STRATEGY: (population) (men) (gender Norms/stereotypes) (sexual/reproductive health)**

Scopus: 3, 007 results [Nov 14 2023]

ProQuest: 810 [Nov 13 2023]

PubMed: 2, 591 [Nov 13 2023]

Embase: 4060 [Nov 14 2023]

Web of Science: 3, 367 [Nov 13 2023]

PsycINFO: 2176 [Nov 14 2023]

CINHL: 13, 287 [Nov 13 2023]

Emcare: 1148 [Nov 14 2023]

**LOGIC GRID: SCOPUS**

| **CALD/migrants** | **Men** | **Gender** | **Sexual and reproductive health** |
| --- | --- | --- | --- |
| (refugee* OR  “asylum seeker*” OR  migrant* OR  “ethnic minority” OR  “culturally and linguistically diverse OR  immigrant*” OR  CALD OR  emigrant* OR  “Ethnic group” OR  “Minority group*” OR  transient).ti,ab | (men OR  male OR  man OR  boy* OR  youth* OR  father OR  paternal).ti,ab | ("gender norm*" OR  "gender stereotype" OR "gender identity" OR  "gender role*" OR  "gender equality" OR  "gender inequality" OR "gender difference*" OR  "gender discrimination" OR  "sexist belief" OR  "traditional values" OR traditions OR  masculinity OR  stigma OR  relationship ).ti,ab, key word | (sexuality OR  "sexual activity" OR  sex OR  "sexual intercourse" OR  "sexual health" OR  "sexual conflict" OR  "reproductive health" OR  "sexual practice*" OR  "sex* dysfunction" OR   "sexual intimacy" OR  "Sexual Behavio?r" OR  Abortion OR  fertility OR  infertility OR  parenthood OR  "family planning" OR  "psychological sexual dysfunction*" OR  "Sexual Harassment" OR  "sexual desire" OR  "sexual pain" OR  "sexual satisfaction" OR  "Sexual health care" OR  "Sexual health service” OR  psychosexual OR  "Sexual health clinic" OR  "Sexual Disorder*" OR  "Sexual Abstinence" OR  "HIV" OR  "Sexually Transmitted Diseases" OR  "Sexually Transmitted Diseases" OR  "unprotected sex" OR  std OR  sti OR  "Human immunodeficiency virus" OR   aids OR   "Unsafe Sex" OR  "Sex Education" OR  "Sexual Trauma" OR  "Religion and Sex" OR   "Sexual and Gender Minorities" OR  "Sex Workers" OR  condoms OR   "sexual experience" OR  "early sexual debut" OR  "sexual initiation" OR  "sexual well-being" OR  "sexual coercion" OR   "sexual abuse" OR  Abortion OR   "sexual healthcare utilization" OR   "Health Services Accessibility" OR  "Community Health Centers" OR   "health access” OR  Acculturation ).ti,ab, keyword |

Results: 2, 718 results (2000-current) (November 14, 2023)

**LOGIC GRID: PubMed**

| **CALD/migrants** | **Men** | **Gender** | **Sexual and reproductive health** |
| --- | --- | --- | --- |
| “refugee*”[mh] OR “transients and migrants” [mh] OR  refugee*[tiab] OR asylum seeker*[tiab] OR migrant*[tiab] OR ethnic minorit*[tiab] OR "culturally and linguistically diverse"[tiab] OR immigrant*[tiab] OR CALD[tiab] OR “Ethnic group*”[tiab] OR Minority group*[tiab] 128,965 | men [mh] OR  male [mh] OR  man [tiab] OR  boy* [tiab] OR  youth* [tiab]OR  father* [mh] OR  paternal behavio?r [mh] OR  young adult [mh] OR  adolescen* [mh]  10, 603, 143 | gender equity [mh] OR masculinity [mh] OR social stigma [mh] OR stereotyping [mh] OR social norm* [mh] OR gender identit* [mh] OR gender role* [mh] OR “sexual and gender minority” [mh] OR acculturation [mh]  gender norm* [tiab] OR gender stereotyp* [tiab] OR gender identit* [tiab] OR gender role* [tiab] OR gender equality [tiab] OR gender inequality [tiab] OR gender equity [tiab] OR gender difference* [tiab] OR gender discrimination [tiab] OR sexist belief*[tiab] OR traditional values [tiab] OR traditions [tiab] OR masculinity [tiab] OR social stigma [tiab] OR family relationship* [tiab] OR stereotyping [tiab] OR social norm* [tiab] OR “sexual and gender minority” [tiab] OR acculturation [tiab]  131, 366 | sexuality [mh] OR sex [mh] OR sexual health [mh] OR reproductive health [mh] OR sexual Behavio?r [mh] OR abortion, induced [mh] OR fertility [mh]OR infertility [mh] OR Sexual Harassment [mh] OR Disorders of Sex Development [mh] OR HIV Infections [mh] aids OR HIV [mh] OR Family planning [mh] OR Unsafe Sex [mh] OR Sex Education [mh] OR Sexual Trauma [mh] OR Religion and Sex [mh] OR marriage [mh] OR Sexual and Gender Minorities [mh] OR condoms [mh] OR contraceptive devices, male [mh] OR Puberties, Precocious [mh] OR Community Health Center* [mh] OR psychosexual development [mh]  OR sexual activity [tiab] OR sexual intercourse [tiab] OR sexual conflict [tiab] OR sexual practice [tiab] OR sex dysfunction[tiab] OR sexual intimacy[tiab] OR parenthood [tiab] OR family planning[tiab] OR Sexual Dysfunctions OR sexual desire OR sexual satisfaction OR Sexual health care OR Sexual Disorder [tiab] OR sexually Transmitted Diseases[tiab] OR unprotected sex[tiab] OR std[tiab] OR sti[tiab] OR Human immunodeficiency virus[tiab] OR Sexual risk behavio?r[tiab] OR sexual experience[tiab] OR sexual initiation[tiab] OR sexual well-being[tiab] OR sexuality [tiab] OR sex [tiab] OR sexual health [tiab] OR reproductive health [tiab] OR sexual Behavio?r [tiab] OR abortion, induced [tiab] OR fertility [tiab]OR infertility [tiab] OR Sexual Harassment [tiab] OR Disorders of Sex Development [tiab] OR HIV Infections [tiab] aids OR HIV [tiab] OR Family planning [tiab] OR Unsafe Sex [tiab] OR Sex Education [tiab] OR Sexual Trauma [tiab] OR Religion and Sex [tiab] OR marriage [tiab] OR Sexual and Gender Minorities [tiab] OR condoms [tiab] OR contraceptive devices, male [tiab] OR Puberties, Precocious [tiab] OR Community Health Center* [tiab] OR psychosexual development [tiab]  502, 782 |

1. “refugee*”[mh] OR “transients and migrants” [mh] OR

refugee*[tiab] OR asylum seeker*[tiab] OR migrant*[tiab] OR ethnic minorit*[tiab] OR "culturally and linguistically diverse"[tiab] OR immigrant*[tiab] OR CALD[tiab] OR “Ethnic group*”[tiab] OR Minority group*[tiab]

Results: 128, 965

AND

1. men[mh] OR male[mh] OR man OR boy OR father*[mh] OR paternal behavior[mh] OR young adult[mh] OR adolescen*[mh] OR men[tiab] OR male[tiab] OR man OR boy OR father*[tiab] OR paternal behavio?r[tiab] OR young adult[tiab] OR adolescen*[tiab]

Results: 10, 603, 143

AND

1. gender equity [mh] OR masculinity [mh] OR social stigma [mh] OR stereotyping [mh] OR social norm* [mh] OR gender identit* [mh] OR gender role* [mh] OR “sexual and gender minority” [mh] OR acculturation [mh] OR

gender norm* [tiab] OR gender stereotyp* [tiab] OR gender identit* [tiab] OR gender role* [tiab] OR gender equality [tiab] OR gender inequality [tiab] OR gender equity [tiab] OR gender difference* [tiab] OR gender discrimination [tiab] OR sexist belief*[tiab] OR traditional values [tiab] OR traditions [tiab] OR masculinity [tiab] OR social stigma [tiab] OR family relationship* [tiab] OR stereotyping [tiab] OR social norm* [tiab] OR “sexual and gender minority” [tiab] OR acculturation [tiab]

Results: 131, 366

AND

1. sexuality[mh] OR sexual activity[tiab] OR sex[mh] OR sexual intercourse[tiab] OR sexual health[mh] OR sexual conflict[tiab] OR reproductive health[mh] OR sexual practice[tiab] OR sex dysfunction[tiab] OR sexual intimacy[tiab] OR sexual Behavior[mh] OR abortion[tiab] OR fertility[mh]OR infertility[mh] OR parenthood[tiab] OR family planning[tiab] OR Sexual Dysfunctions[tiab] OR Sexual Harassment[mh] OR sexual desire[tiab] OR sexual satisfaction[TIAB] OR Sexual health care[tiab]OR psychosexual development[mh] OR Sexual Disorder[tiab] OR Disorders of Sex Development[mh] OR HIV Infections[mh] OR sexually Transmitted Diseases[tiab] OR unprotected sex[tiab] OR std[tiab] OR sti[tiab] OR Human immunodeficiency virus[tiab] OR aids[tiab] OR HIV[mh] OR Family planning[mh] OR Sexual risk behavio?r[tiab] OR Unsafe Sex[mh] OR Sex Education[mh] OR Sexual Trauma[mh] OR Religion and Sex[mh] OR marriage[mh] OR Sexual and Gender Minorities[mh] OR condoms[mh] OR contraceptive devices, male[mh] OR sexual experience[tiab] OR Puberties, Precocious[mh] OR sexual initiation[tiab] OR sexual well- being[tiab] OR Community Health Centers[mh] OR acculturation[tiab]
2. 1 AND 2 AND 3 AND 4: 3, 024
3. 5 restricted to english language and (yr= 2000-current): **2, 592 [Nov 20 2023]**

**EMBASE:**

| CALD/migrants | Men | Gender | Sexual and reproductive health |
| --- | --- | --- | --- |
| exp *migration/ OR  exp *Refugee.sh. OR  exp vulnerable population/ OR  (Ethnic group* OR "Culturally and Linguistically Diverse" OR OR Minority group* OR Asylum seeker* OR Displaced people OR Immigrant* OR Emigrant* OR asylum OR ethnic minorit* OR Transient*) | exp *male/ OR exp boy/ OR exp father/ OR exp juvenile/ OR exp paternal behavio?r/ OR exp young adult/ OR exp adolescen*/ OR exp lgbtqia+ people/  OR  (youth/) | exp *social norm/ OR  exp stereotyping/ OR  exp gender identity/ OR  exp gender equity/ OR  exp masculinit/ OR  exp social stigma/ OR  exp family relation*/ OR  exp “sexual and gender minority”/ OR  exp attitude to sexuality/  OR  (gender norm*/ OR  gender stereotyp*/ OR  gender equality/ OR  gender inequality/ OR  gender difference*/ OR  gender discrimination/ OR  sexist belief*/ OR  traditional values/ OR  traditions) | exp *sexuality/ OR  exp sex/ OR  exp *“gender and sex”/ OR  exp sexual intercourse/ OR  exp sexual health/ OR  exp reproductive health/ OR  exp sex dysfunction/ OR  exp sexual Behavio?r/ OR  exp abortion/OR  exp attitude to abortion/ OR  exp fertility/ OR  exp male fertility/ OR  exp infertility/ OR  exp male infertility/ OR  exp parenthood/ OR  exp family planning/ OR  exp psychosexual Disorder/ OR  exp Sexual Harassment/ OR  exp psychosexual development/ OR  exp sexual dysfunction/ OR  exp human immunodeficiency virus Infection*/ OR  exp sexually transmitted diseases/ OR  exp unprotected sex/ OR  exp acquired immune deficiency syndrome/ OR  exp Family planning/ OR  exp Unsafe Sex/ OR  exp safe sex/ OR  exp sex Education/ OR  exp Sexual Harassment/ OR  exp marriage/ OR  exp condom*/ OR  exp puberty/ OR  exp sexual well-being/ OR  exp sexual abuse/ OR  exp Health Disparity/ OR  exp health centre/ OR  (health access/ OR  sexual activity/ OR  sexual conflict/ OR  Community Health Centers/ OR  sexual initiation/ OR  sexual healthcare utilization/ OR  “Religion and Sex”/OR  std/ OR  sti/ OR  aids/ OR  Sexual health clinic/ OR  Sexual Disorder/ OR  Sexually Transmitted Diseases/ OR  Sexual Dysfunctions/ OR  sexual satisfaction/ OR  Sexual health care/ OR  Sexual risk behavio?r/ or  Acculturation/) |

1. (exp migration or exp Refugee or vulnerable population)

Results: 50649

OR

1. (migration or Refugee or vulnerable population or Ethnic group* or "Culturally and Linguistically Diverse" or Minority group* or Asylum seeker* or Displaced people or Immigrant* or Emigrant* or asylum or ethnic minorit* or Transient*).ti,ab.

Results: 991864

**3. 1 OR 2: 10, 44208**

AND

4. (exp male or exp boy or exp father or exp paternal behavio?r or exp lgbtqia+ people)

Results: 13335931

OR

5. (male or men or boy or father or lgbtqia+ people or paternal behavio?r).ti,ab.

Results: 2149454

**6: 4 OR 5: 12253486**

AND

7. (exp social norm or exp stereotyping or exp gender identity or exp gender equity or exp masculinity or exp social stigma or exp family relation or exp "sexual and gender minority" or exp attitude to sexuality)

Results: 82926

OR

8. (social norm or stereotyping or gender identity or gender equity or masculinity or social stigma or family relation or gender norm or “sexual and gender minority” or attitude to sexuality or gender norm or gender stereotyp* or gender equality or gender inequality or gender difference* or gender discrimination or sexist belief* or traditional values or traditions).ti,ab

Results: 82652

**9: 7 OR 8: 2774362**

AND

10. (exp sexuality or exp sex or exp "gender and sex" or exp sexual intercourse or exp sexual health or exp reproductive health or exp sex dysfunction or exp sexual Behavio?r or exp abortion or exp attitude to abortion or exp fertility or exp male fertility or exp infertility or exp male infertility or exp parenthood or exp family planning or exp psychosexual disorder or exp Sexual Harassment or exp psychosexual development or exp sexual dysfunction or exp human immunodeficiency virus Infection or exp sexually transmitted diseases or exp unprotected sex or exp acquired immune deficiency syndrome or exp Family planning or exp Unsafe Sex or exp safe sex or exp sex Education or exp Sexual Harassment or exp marriage or exp condom or exp puberty or exp sexual well-being or exp sexual abuse or exp Health Disparity or exp health centre)

Results: 1265151

OR

11. (health access or sexual activity or sexual conflict or Community Health Centers or sexual initiation or sexual healthcare utilization or "Religion and Sex" or std or sti or aids or Sexual health clinic* or Sexual disorder or Sexually Transmitted Diseases or Sexual Dysfunction* or sexual satisfaction or Sexual health care or Sexual risk behavio?r or sexualit* or sex or "gender and sex" or sexual intercourse or sexual health or reproductive health or sexual Behavio?r or abortion or attitude to abortion or fertility or male fertility or infertility or male infertility or parenthood or family planning or psychosexual disorder or Sexual Harassment or psychosexual development or human immunodeficiency virus Infection* or sexually transmitted disease* or unprotected sex or acquired immune deficiency syndrome or Family planning or Unsafe Sex or safe sex or sex Education or Sexual Harassment or marriage or condom* or puberty or sexual well-being or sexual abuse or Health Disparity or health centre).ti,ab

Results:

12: 10 OR 11: 2935870

13: 3 AND 6 AND 9 AND 12: 4190 [Nov 16]

14: 13 restricted to (english language and yr= “2000-current”): **3940 [Nov 202023]**

**EMCARE:**

1 OR 2:  **185, 257**

4 OR 5: **1958,729**

7 OR 8: **107, 610**

10 OR 11: **710, 712**

3 AND 6 AND 9 AND 12: **1854**

(restricted to english language and yr= “2000-current”): **1770 [Nov 202023]**

**CINAHL Complete (EBSCOhost):**

(MM “refugees+”) OR (MM “Minority group*+”) OR (MM “immigrants”) OR (MM “emigration and immigration”) OR (MM "cultural diversity”) OR (MM "cultural diversity") OR (MM “Emigrant*”) OR (MM “transients and Migrants*”) OR (AB= “refugees+” OR “racial and Ethnic group*+” OR "cultural diversity” OR “ethnic groups” OR “Minority group*+” OR “immigrant*+” OR "cultural diversity" OR “Asylum seeker*+” OR "racial and ethnic Groups+” OR "racial and ethnic differences+” OR “vulnerable population+” OR "Culturally and Linguistically Diverse+" OR “Emigrant*” OR “Migrant*” OR “ethnic minorit*” OR “transients and Migrants”)

1. Results: 33, 045

7, 431

MM (“male*+”) OR MM (“fathers+”) OR (MM “lgbtq+ persons”+) OR (AB= “boy” OR “paternal” OR “male” OR “men”)

2. Results: 816, 652

(MM “cultural diversit+”) OR (MM “gender identity+”) OR (MM “cultural sensitivity+”) OR (MM “social norm*”) OR (MM “stereotyping”) OR (MM “attitude to sexuality”) OR (MM “Gender role”) +” OR (MM “masculinity”) OR (MM “Sexis+”) OR (MM “stigma”) OR (MM “family relations*”) OR (MM “discrimination”) OR (MM “sexual and gender minorities”) OR (MM “parental attitudes”) OR (MM “gender equality+”) OR (MM “gender role”) OR (MM “social values+”) OR (MM “traditions”) OR (MM “acculturation”) OR

AB=“cultural diversit+” OR “gender identity+” OR “cultural sensitivity” OR “social norm*” OR “stereotyping” OR “sex discrimination” OR “sexis*” OR “masculinity” OR “Gender role” OR “attitude to sexuality” OR “social equality” OR “social equity” OR “stigma” OR “family relations” OR “social discrimination” OR “ethnic discrimination” OR “sexual and gender minorities” OR “parental attitudes” OR “gender equality+” OR “gender role” OR “ethnic values” OR “social values” OR “sociocultural factors+” OR “gender norm” OR “gender stereotype” OR “gender inequality” OR “traditional values” OR “acculturation”

3. Results: 14, 827

(MM “socioeconomic disparities in health”) OR (MM “sexuality+”) OR (MM “sex”) OR (MM “unsafe sex”) OR (MM “sexual dysfunction, male”) OR (MM “sexual health”) OR (MM “reproductive health+”) OR (MM “attitude to abortion”) OR (MM “abortion induced”) OR (MM “fertility”) OR (MM “infertility”) OR (MM “family planning”) OR (MM “adolescent development”) OR (MM “Sexual Harassment”) OR (MM “sexually transmitted diseases”) OR (MM “sex education”) or ( MM “Human Immunodeficiency Virus”) OR (MM “safe sex”) OR (MM “sexual abuse+”) OR (MM “contraception”) OR (MM “puberty”) OR ( MM “healthcare disparities”) OR (MM “Community Health Centre”) OR (MM “sexual behavio?r”)

OR (AB= male fertility OR psychosexual disorder OR sexual trauma OR Condom* OR sexual conflict OR sexual intimacy OR “Religion and Sex” OR std OR sti OR sexual well-being OR sexual coercion OR unprotected sex OR Sexual risk behavio?r* OR Sexual practice* OR “sociocultural factors+” OR “health care services” OR “socioeconomic disparities in health”) OR “sexuality+” OR “sexual behavio?r” OR “sex” OR “sexual reproduction” OR “sexual dysfunction” OR “sexual health” OR “reproductive health+” OR “attitude to abortion” OR “abortion” OR “fertility” OR “infertility” OR “family planning” OR “psychosexual behavio?r” OR “adolescent development” OR “sexually transmitted diseases” OR “health care utilization” OR “Human Immunodeficiency Virus” OR “HIV” OR “AIDS” OR “contraception” OR “family planning” OR “sexual satisfaction” OR “health care services” OR “Community Health Centre” OR “sexual behavio?r”)

Results: 591, 968

1 AND 2 AND 3 AND 4: 313, 060

5 restricted to English language and (yr=2000-current): **311, 701 [Nov 16 2023]**

**Web of Science:**

((TOPIC=(refugee* OR “asylum seeker*” OR migrant* OR “ethnic minorit*” OR "culturally and linguistically diverse" OR immigrant* OR CALD OR "Emigrants and Immigrants" OR “displaced people” OR "Sexual and Gender Minorities” OR "Transients and Migrants” OR “Ethnic group*” OR “Minority group*”))

AND

TOPIC=(men OR male OR man OR boy* OR father OR paternal ))

AND

TOPIC=("gender norm*" OR "gender stereotyp*" OR "gender identit*" OR "gender role*" OR "gender equality" OR "gender inequality" OR "gender difference*" OR "gender discrimination" OR Sexis* OR "traditional values" OR traditions OR masculinit* OR stigma OR relationship* OR acculturation))

AND

TOPIC=(sexuality OR "sexual activity" OR sex OR "sexual intercourse" OR "sexual health" OR "sexual conflict" OR "reproductive health" OR "sexual practice*" OR "sex* dysfunction" OR "sexual intimacy" OR "Sexual Behavio?r" OR abortion OR fertility OR infertility OR parenthood OR "family planning" OR "Sexual Harassment" OR "Sexual health care" OR psychosexual OR "Sexual health clinic" OR "HIV" OR "Sexually Transmitted Disease*" OR "unprotected sex" OR std OR sti OR "Human immunodeficiency virus" OR aids OR "Family planning" OR "Sexual risk behavio?r*" OR "Unsafe Sex" OR "Sex Education" OR "Sexual Trauma" OR "Religion and Sex" OR marriage OR condoms OR "sexual initiation" OR "sexual well-being" OR abortion OR "sexual healthcare utilization" OR "Health Services Accessibility" OR "Community Health Centers" OR "health access”)

Results: **2, 689 (2000-2023) (November 20, 2023)**

**ProQuest HMS database only**

TI,AB(refugee* OR "asylum seeker*" OR migrant* OR "ethnic minorit*" OR "culturally and linguistically diverse" OR immigrant* OR cald OR "Transients and Migrants" OR "Emigrants and Immigrants" OR "Ethnic group*")

AND

TI,AB(men OR male OR man OR boy* OR youth* OR father OR paternal OR young OR adolescen*)

AND

TI,AB("gender norm*" OR "gender stereotyp*" OR "gender role*" OR "gender equality" OR "gender inequality" OR "gender difference*" OR "gender discrimination" OR Sexis* OR "traditional values" OR traditions OR masculinit* OR stigma OR relationship* OR acculturation)

AND

TI,AB(sexuality OR "sexual activity" OR sex OR "sexual intercourses" OR "sexual health" OR "sexual conflict" OR "reproductive health" OR "sexual practice*" OR "sex* dysfunction" OR "Sexual Behavior" OR abortion OR fertility OR infertility OR parenthood OR "family planning" OR "Sexual Dysfunctions" OR "Sexual Harassment" OR "Sexual health care" OR "Sexual health service" OR psychosexual OR "Sexual Disorder*" OR HIV OR "Sexually Transmitted Diseases" OR "unprotected sex" OR std OR sti OR "Human immunodeficiency virus" OR aids OR "Sexual risk behavior*" OR "Unsafe Sex" OR "Sex Education" OR "Sexual Trauma" OR "Religion and Sex" OR marriage OR "Sexual and Gender Minorities" OR condoms OR "sexual initiation" OR "sexual well-being" OR "sexual abuse" OR abortion OR "Delivery of Health Care" OR "Community Health Centers" OR "health access")

Results: **812** (full-text, peer-reviewed 2000-2023) (November 20, 2023)

**PsychINFO:**

| **CALD/migrants** | **Men** | **Gender** | **Sexual and reproductive health** |
| --- | --- | --- | --- |
| exp refugees/ OR exp "racial and Ethnic group*"/ OR exp Minority group*/ OR exp immigration/ OR exp Asylum seeker*/ OR exp "racial and ethnic Groups"/ OR exp "racial and ethnic differences"/ OR (vulnerable population/ OR "Culturally and Linguistically Diverse"/ OR Immigrant*/ OR Emigrant*/ OR Migrant*/ OR ethnic minorit*.mp.) | exp human males/ OR exp fathers/ OR  exp emerging adulthood/ OR  exp lgbtq/  exp sexual Orientation/ OR  (boy/ OR juvenile/ OR paternal/ OR young adult/ OR adolescen*/ OR  OR youth) | exp cross cultural differences/ OR exp gender identity/ OR exp cultural sensitivity/ OR exp *social norm/ OR exp stereotyped attitudes/ OR exp sex discrimination/ OR exp sex role attitudes/ OR exp social equality/ OR exp social equity/ OR exp masculinit/ OR exp Sexis*/ OR exp stigma/ OR exp family relations*/ OR exp social discrimination/ OR exp racism/ OR exp "racial and ethnic discrimination"/ OR exp sexual minority groups/ OR exp parental attitudes/ OR exp gender equality/ OR exp human sex differences/ OR exp ethnic values/ OR exp sociocultural factORs/ OR exp authORitarianism/ OR (gender norm*/ OR gender stereotyp*/ OR gender inequality/ OR traditional values/ OR traditions/ OR gender equity/ OR acculturation) | exp sociocultural Factors/ OR exp health care services/ OR exp health disparities/ OR exp sexual attitudes/ OR exp multiculturalism/ OR exp acculturation/ OR exp *sexuality/ OR exp sex/ OR exp sex role attitudes/ OR exp sexual reproduction/ OR exp sexual health/ OR exp reproductive health/ OR exp sexual function disturbances/ OR exp "abortion(attitudes toward)"/ OR exp abortion laws/ OR exp fertility/ OR exp infertility/ OR exp family planning/ OR exp psychosexual behavior/ OR exp adolescent development/ OR exp Sexual Harassment/ OR exp sexually transmitted diseases/ OR exp health care utilization/ OR exp sexual risk taking/ OR exp HIV/ OR exp sex education/ OR exp AIDS/ OR exp sexually transmitted diseases/ OR exp AIDS prevention/ OR exp safe sex/ OR exp AIDS/ OR exp sexual abuse/ OR exp Sexual Harassment/ OR exp marriage/ OR exp domestic violence/ OR exp condoms/ OR exp family planning attitudes/ OR exp puberty/ OR exp sexual satisfactions/ OR exp sexual abuse/ OR exp healthcare utilization/ OR exp health care costs/ OR exp health care services/ OR exp Health disparities/ OR exp Community Health/ OR (sexual intercourse/ OR sexual Behavior/ OR abortion/ OR male fertility/ OR psychosexual Disorder/ OR sexual trauma/ OR condom*/ OR sexual conflict/ OR sexual intimacy/ OR "Religion and Sex"/ OR std/ OR sti/ OR aids/) |

1. (Refugees OR Minority groups OR immigration OR Asylum seeking OR at risk populations).sh

Results: 92895

OR

2. ("racial and Ethnic group" OR Asylum seeker OR "racial and ethnic differences” OR vulnerable population OR “Culturally and Linguistically Diverse” OR migrant OR ethnic minority).ti,ab.

Results: 23481

3. 1 OR 2: 109749

4. (human males OR fathers OR LGBTQ OR adolescent fathers).sh Results: 46163

5. (Males OR men OR boy OR juvenile OR paternal).ti,ab Results: 387, 847

6. 4 OR 5: 424069

7. (cross cultural differences OR gender identity OR cultural sensitivity OR social norms OR stereotyped attitudes OR sex discrimination OR stereotyped attitudes OR sex discrimination OR sex role attitudes social equality OR masculinity OR Sexism OR stigma OR family relations* OR social discrimination OR racism OR "racial and ethnic discrimination" OR sexual minority groups OR parental attitudes OR gender equality OR human sex differences OR ethnic values OR sociocultural Factors OR Authoritarianism).sh

OR

8. (cross cultural differences OR gender identity OR cultural sensitivity OR social norms OR stereotyped attitudes OR sex discrimination OR stereotyped attitudes OR sex discrimination OR sex role attitudes social equality OR masculinity OR Sexism OR stigma OR family relations* OR social discrimination OR racism OR "racial and ethnic discrimination" OR sexual minority groups OR parental attitudes OR gender equality OR human sex differences OR ethnic values OR sociocultural Factors OR Authoritarianism OR gender norm* OR gender stereotyp* OR gender inequality OR traditional values OR traditions OR gender equity OR acculturation).ti,ab

9. 7 OR 8: 42, 3820

10. (sociocultural Factors OR health care services OR health disparities OR sexual attitudes OR multiculturalism OR acculturation OR sexualit* OR sex OR sex role attitudes OR sexual reproduction OR sexual health OR reproductive health OR sexual function disturbances OR "abortion(attitudes toward)" OR abortion laws OR fertility OR infertility OR family planning OR psychosexual behavior OR adolescent development OR Sexual Harassment OR sexually transmitted diseases OR health care utilization OR sexual risk taking OR HIV OR sex education OR AIDS OR sexually transmitted diseases OR AIDS prevention OR safe sex OR sexual abuse OR Sexual Harassment OR marriage OR domestic violence OR condoms OR family planning attitudes OR puberty OR sexual satisfactions OR sexual abuse OR healthcare utilization OR health care costs OR health care services OR Health disparities OR Community Health).sh

OR

11. (sexual intercourse OR sexual Behavior OR abortion OR male fertility OR psychosexual Disorder OR sexual trauma OR condom* OR sexual conflict OR sexual intimacy OR "Religion and Sex" OR std OR sti OR aids OR sociocultural Factors OR health care services OR health disparities OR sexual attitudes OR multiculturalism OR acculturation OR sexualit* OR sex OR sex role attitudes OR sexual reproduction OR sexual health OR reproductive health OR sexual function disturbances OR "abortion(attitudes toward)" OR abortion laws OR fertility OR infertility OR family planning OR psychosexual behavior OR adolescent development OR Sexual Harassment OR sexually transmitted diseases OR health care utilization OR sexual risk taking OR HIV OR sex education OR AIDS OR sexually transmitted diseases OR AIDS prevention OR safe sex OR sexual abuse OR Sexual Harassment OR marriage OR domestic violence OR condoms OR family planning attitudes OR puberty OR sexual satisfactions OR sexual abuse OR healthcare utilization OR health care costs OR health care services OR Health disparities OR Community Health).ti,ab

12. 10 OR 11: 613, 162

13. 3 AND 6 AND 9 AND 12: 1974

14. 13 restricted to english language (yr= “2000-current”): **1625[Nov 20 2023]**
